# Analysis of the Anthocyanin Degradation in Blue Honeysuckle Berry under Microwave Assisted Foam-Mat Drying

**DOI:** 10.3390/foods9040397

**Published:** 2020-03-31

**Authors:** Yu Sun, Yuhan Zhang, Wei Xu, Xianzhe Zheng

**Affiliations:** 1 College of Food Engineering, Harbin University, Harbin 150086, China; yusun2019@163.com (Y.S.); xweihappy@163.com (W.X.); 2 College of Engineering, Northeast Agricultural University, Harbin 150030, China; zyh979462566@163.com

**Keywords:** berries, anthocyanin, degradation, microwave, foam-mat drying, simulation

## Abstract

Changes in nutrient content and bioactivity are important indicators to evaluate the quality of products. Berries are rich in antioxidant anthocyanins, which are prone to degradation during drying. The effects of different variables on the stability of anthocyanins in berry puree during microwave assisted foam-mat drying (MFD) was investigated by path analysis and degradation kinetics analysis. The experimental results showed that the degradation of anthocyanins mainly occurred in the last drying stage. The temperature and the moisture content have both direct and indirect effects on the anthocyanin stability. The direct path coefficient of the moisture content on anthocyanins was 0.985, and the direct path coefficient of temperature on anthocyanins was −0.933. The moisture content to temperature ratio (*M/T*) was first put forward to estimate the anthocyanin degradation. The results of the regression analysis confirmed that the anthocyanins were stable at *M/T* of 0.96–3.60. A finite element simulation model was established to predict the anthocyanin degradation rate and content. These research results could provide a theoretical reference for use in optimizing the MFD processing technologies.

## 1. Introduction

Berries are well known for their unique flavor and abundant nutrients, including organic acids, vitamins, sugars, and trace elements, and especially for their large quantity of antioxidants [1]. Anthocyanins are the main source of antioxidants in berries. Anthocyanins are helpful to reduce cholesterol concentration, alleviate high blood pressure, and improve eyesight. Berries have both nutritional and health functions [2]. Thus, the Food and Agriculture Organization of the United Nations positioned the berry as a third-generation fruit with health-care functions [3]. The storage and transportation of berries are quite difficult because they are juicy, fragile, and limited seasonal fruits. Therefore, berries are often processed into products such as jam, juice, and powder [4]. China has abundant berry resources; the area of berry cultivation is over 45,000 hectares, and the annual output is more than 300,000 tons [5]. Currently, less than 30% of the total yield of fresh berries are processed into products, so there is a large market space for processed berry products [6].

The shelf life of fresh berries is 3–4 days at room temperature, due to their moisture content of over 90% (*w.b.*) [7]. In addition to the necessary refrigeration and freezing, drying is one of the most effective methods for the processing and storage of harvested berries [8]. Conventional hot air drying methods are not suitable for berries [9]. When dried in high temperature and oxygen conditions for a long time the bioactive ingredients in berries degrade massively and the quality of the product decreases [10]. Freeze dried products usually have fine quality. However, this process takes a long time, approximately about 24–48 h, which leads to the increase of production costs [11]. Spray drying is the primary method for producing powders; however, the uniformity of droplets is poor due to the high viscosity of berry puree [12]. Foam-mat drying is an adaptive method for viscous, sticky, and heat sensitive materials [13]. In this drying process, the liquid material is converted into a foam material by being whipped after adding an edible foaming agent [14]. As foam-mat drying is less expensive and less complicated, it has been used to dry mango, papaya, banana, and so on. [15,16,17]. Foam-mat drying combined with microwave heating shortens the drying duration and protects the thermally susceptible nutrients more than when combined with hot air drying, as microwave energy can be instantaneously converted into volumetric heating [18,19]. The heat transfer and mass transfer during microwave drying have the same transfer direction from the inside to the outside, which produces high heating speed and a high energy utilization rate [20]. Microwave-assisted foam-mat drying (MFD) technology makes full use of the advantages of microwave drying and foam-mat drying and is a suitable drying method for berries, including blueberries, raspberries, and other fruits [21].

The contents and bioactivity of nutrients are important indicators for evaluating the quality of products. Anthocyanins, as a class of polyphenolic pigments, are widely present in plant organs, and they have excellent water solubility and bioactivity [22]. However, the anthocyanin pigments are unstable when the processing parameters, such as the temperature, pH, oxygen content, and light, change [23,24]. The anthocyanin degradation was the maximum in neutral environment at 75 °C, and the degradation was found to follow the first-order kinetics [25]. Similar results indicated that anthocyanins were stable below 60 °C, whereas they tended to degrade at temperatures higher than 80 °C [26]. Furfural compounds generated by the thermal degradation of sugars also promoted the degradation of anthocyanins [27]. Existing studies have focused on the relationship between various single variables and the degradation of anthocyanins, while few studies have comprehensively considered all of the processing factors regarding the stability of anthocyanins during the drying process.

This study aimed to determine the mechanism of anthocyanin degradation in berry puree by considering multiple variables during the MFD. Based on the experimental results in different microwave intensities, path analysis has been implemented to study the effects of multiple variables (temperature, moisture content, drying time and radius) on the anthocyanin degradation content. A multi-physical simulation model was established by using the results of degradation kinetic analysis, which could be a theoretical and technical reference for product quality control.

## 2. Materials and Methods

### 2.1. Preparation of Foamed Berry Puree

**Figure 1:**
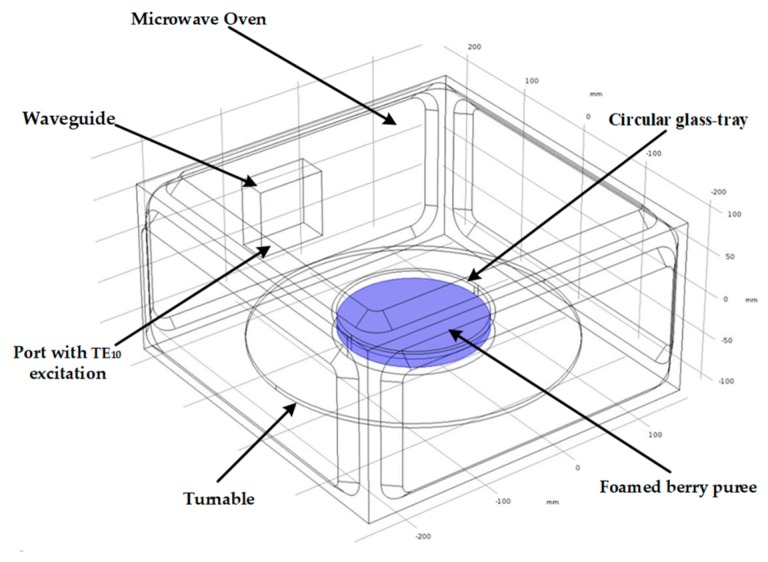
Schematic of the microwave oven.

The drying of blue honeysuckle puree was carried out in a microwave workstation (Figure 1). Fresh blue honeysuckle (*Lonicera caerulea* L.) berries were selected according to their color and maturity (provided by the horticulture station of Northeast Agricultural University, Harbin, China). The uniform and fine berry puree was prepared by grinding in a mixer (JYL-Y5, Jiuyang Ltd., Jinan, China) after removing impurities, cleaning, and wiping off the surface water. The optimal proportion of the foaming agent was 6 g glycerin monostearate (GMS, Jiashili Co., Ltd., Guangzhou, China) and 3 g soy protein isolate (SPI, Linyi Co., Ltd., Linyi, China) dissolved in 100 mL distilled water, and 10 mL of 0.5% carboxymethyl cellulose (CMC, Zhiyuan Co., Ltd., Tianjin, China) was added as a stabilizer. Foaming agent and berry puree were then mixed with a mass ratio of 1: 1. The mixture was stirred evenly with an electric stirrer for 6 min and stored in a water bath (HWS24, Yiheng Co., Ltd., Shanghai, China) at 70 °C for 30 min to generate homogenous foamed berry puree. Foamed berry puree with the density of 600 kg/m^3^ has been reported to have the best stability [28].

### 2.2. Experimental Process

A circular glass-tray containing 100 g of foamed berry puree was loaded on a turntable in the center of a microwave oven. The microwave oven (NN-ST780, Panasonic, Osaka, Japan) was able to supply 10 accurate power levels with a maximum of 1100 W at 2.45 GHz frequency, with internal dimensions of 395 mm (W) × 180 mm (H) × 385 mm (D). The excitation for the microwave oven was through a rectangular waveguide, which was installed on the right wall of the cavity with TE_10_ as the dominant mode of the transverse electric (TE) waves. The deviation between actual power and rated power was less than 5%, so the rated power was used for calculating the power absorbed by the berry puree in the simulation. The radius of the glass-tray was 8 cm. The temperature, moisture content and anthocyanin content of the berry puree were measured per minute in different positions (radius of 0, 2, 4, 6, 8 cm) when the microwave intensity was set to 6, 7, 8, 9 and 10 W/g. The drying experiments were continued until the moisture content of berry puree was below 15%. Blue honeysuckle powder was obtained by grinding the dried product.

### 2.3. Determination of Variables

#### 2.3.1. Temperature

An infrared camera (T-420, FLIR Systems Inc., Wilsonville, OR, USA) was used to capture the temperature distribution of the foamed berry puree. FLIR Tools software was used to analyze the infrared image and collect the temperature data of the berry puree. In order to ensure that the distance between the camera and the material was unchanged, the infrared camera was fixed on a tripod. The shooting was completed within 3–4 s to minimize the error caused by the operations.

#### 2.3.2. Moisture Content

The moisture content was determined by the direct drying method (GB 5009.3-2010), and was calculated as the ratio of free water content to the whole weight. The moisture content (%) values were calculated using Equation (1). Dried samples of 2–5 g (0.0001 g) were loaded into an aluminum box and then they were put into a drying oven at 105 °C for 3–4 h until the difference of weight between adjoining samples was less than 2 mg.

(1) $$M=\frac{m_{1}-m_{2}}{m_{1}}\times 100\%$$

where *M* is the moisture content (%), *m*_1_ is the weight before drying (g) and *m*_2_ is the constant weight (g).

#### 2.3.3. Total Anthocyanin Content

The total anthocyanin contents were measured by the low concentration of vanillin hydrochloric acid method [29]. 1–2 g of the dried samples were dissolved in 30 mL of methanol solution. The mixture was centrifuged at 4000 rad/min (LG10-2.4A, Jingli Co., Ltd., Beijing, China). After 15 min of centrifugation, 5 mL chromogenic reagent was added into 1 mL supernatant. The chromogenic reagent was a mixture of 1% (*w/v*) vanillin methanol solution and 8% (*w/v*) hydrochloric acid methanol solution in a ratio of 1:1. Then, the tube was transferred to a water bath at 30 °C for 30 min. The absorbance of the solution was determined at 520 nm. The concentration of anthocyanin was calculated by using a calibration curve of standard pro-anthocyanin solutions. The anthocyanin content in the berry puree was calculated by using Equation (2).

(2) $$C=\frac{DVn}{W}$$

where *C* is the anthocyanin content (mg/g), *V* is the constant volume (mL), *D* is the concentration of anthocyanin (mg/mL), *n* is the dilution multiple and *W* is the weight of sample (g).

### 2.4. Statistical Analysis

Regression analysis was performed by Sigmaplot 12.5 (Systat Software Inc., San Jose, CA, USA). The statistical differences among samples were determined by the analysis of variance (ANOVA). The calculation of path analysis was performed by SPSS 24.0 (IBM, Armonk, NY, USA). The multiphysics model of the drying process was simulated by Comsol (Comsol Inc., Stockholm, Stockholm Lan, Sweden). All the data were expressed as mean values of triplicate experiments.

## 3. Results and Discussion

### 3.1. Drying Characteristics of Berry Puree under Microwave Drying Conditions

**Figure 2:**
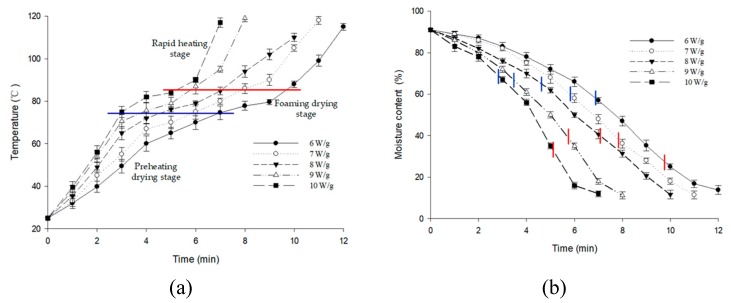
The characteristics of (**a**) temperature and (**b**) moisture content of the berry puree drying at varying microwave intensities. The blue line in (**a**) is the isotherm of 75 °C and the red line in (**a**) is the isotherm of 85 °C. The vertical blue and red lines in (**b**) correspond with (**a**) in the same drying time.

The drying characteristics of the berry puree at varying microwave intensities are given in Figure 2. According to the pattern of changes in the temperature and moisture content of berry puree, the microwave drying process can be divided into three stages: a preheating drying stage, a foaming drying stage, and a rapid heating stage. In the preheating drying stage, the temperature of the berry puree gradually increased from room temperature to 75 °C, while there was a small decrease in the moisture content. Due to the berry puree bubbling during the foaming drying stage, the evaporation was accelerated, and the moisture loss increased remarkably. Meanwhile the temperature fluctuated in the range of 75–85 °C. During the rapid heating stage, the moisture content of the berry puree continued to decrease until the end of drying *(M* < 15%), and the final temperature was close to 120 °C. As can be observed, the drying time was shortened, and the final temperature of the dried product was increased with the increase in microwave intensity [30].

**Figure 3:**
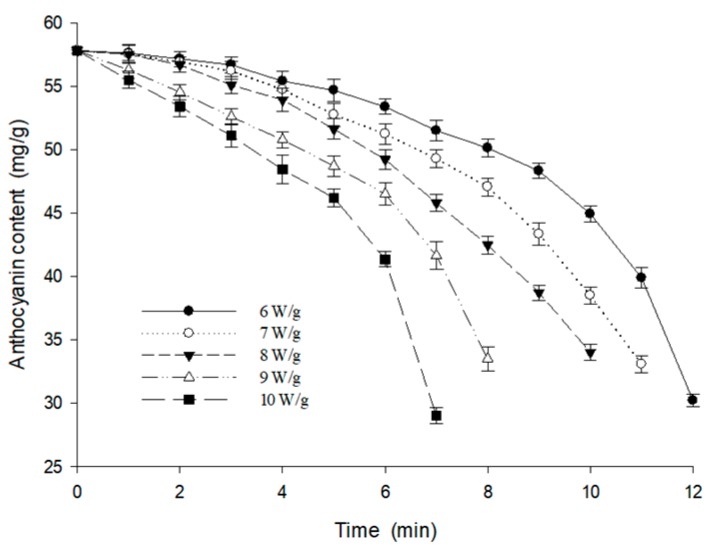
The characteristics of the anthocyanin content in the berry puree drying at varying microwave intensities.

The amount of anthocyanins degradation decreased slowly in the pre-heating drying stage and the early foaming drying stage, as shown in Figure 3. The ratio of anthocyanin degradation reached 30% at the end of the foaming drying stage. This was mainly caused by the high temperature and the decrease of the moisture content in this stage. The tendency was consistent with the results calculated by Verbeyst [31]. Meanwhile, as the moisture content of the berry puree decreased, the concentration of the sugars and acids in the berry puree increased [32]. The increase in the temperature and the concentration of sugars and acids of the berry puree had a synergistic effect on the degradation of anthocyanins [33].

**Figure 4:**
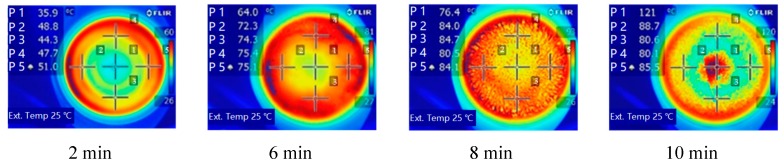
Thermal images of the berry puree at the microwave intensity of 8 W/g.

**Figure 5:**
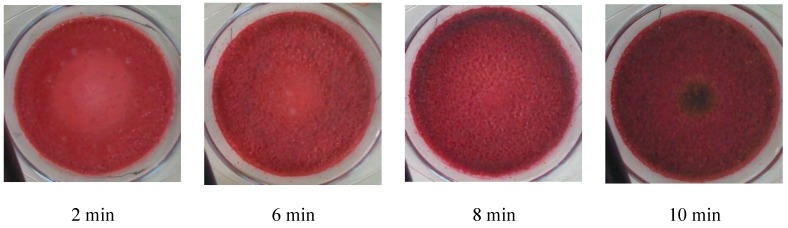
Pictures of the berry puree during drying procedure at the microwave intensity of 8 W/g.

The analysis above illustrates that the microwave intensity influences the stability of anthocyanins through the temperature and moisture content. The anthocyanin degradation was also closely related to the drying time. From the infrared images taken during experiments (Figure 4) and the real-time pictures about the state of berry puree (Figure 5), it can be observed that the drying states of berry puree have obvious differences at the different sampling points. The edges of the loading container heated faster than the center, except for the end of drying.

**Figure 6:**
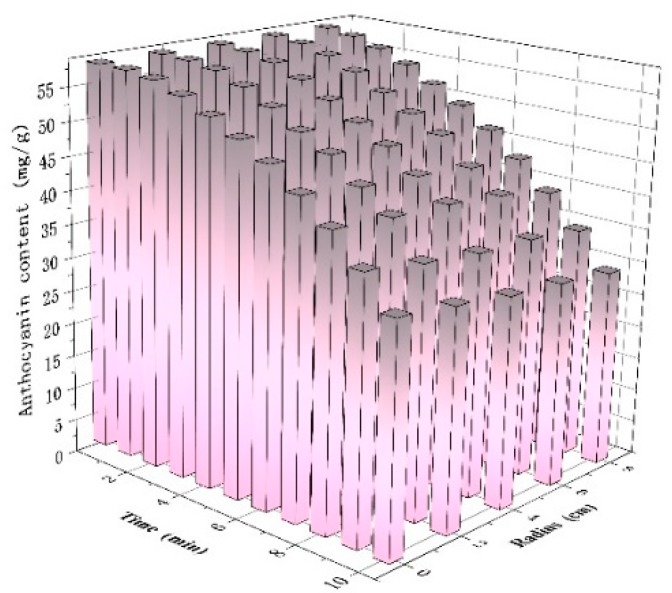
The distribution of anthocyanin content in berry pure at the microwave intensity of 8 W/g.

As the drying stages under the selected microwave intensities showed similar heating patterns, only the intermediate level of microwave intensity was chosen. By fitting the data of the drying time, the radius of the sampling point, and the anthocyanin content, the effects of the drying time and radius on anthocyanin content were clearly shown in Figure 6. The majority of anthocyanins were stable until the drying duration reached 4 min. There was some degradation at the edge regions at 6–8 min. The anthocyanin content decreased sharply after 8 min of drying, and the degradation amount in the central region was higher than in other regions. As illustrated in Figure 4, the central temperature rose to 121 °C at 10 min, which resulted in a large amount of degradation of anthocyanin [34].

### 3.2. Correlation Analysis of Anthocyanin Degradation

#### 3.2.1. Path Analysis of Anthocyanin Degradation

The path analysis method was introduced to reveal the effects of the temperature, moisture content, drying time, and position on the anthocyanin degradation. Path analysis is a multivariate statistical technique proposed by a quantitative geneticist Sewall Wright in 1921 [35]. It is mainly used to analyze the effects of multiple independent variables on a dependent variable. Path analysis analyses the direct and indirect effects of the independent variables on the dependent variable by decomposing the correlation between them. The path analysis method decomposes the correlations between the independent variables and the dependent variable into direct effects and indirect effects, as the simplified correlation coefficient (*r_iy_*) of the independent variable (*x_i_*) on the dependent variable (*y*) = direct path coefficient (*P_iy_*) of *x_i_* on *y* + all the indirect path coefficients of *x_i_* on *y*, where the indirect path coefficients of *x_i_* on *y* = correlation coefficient (*r_ij_*) × direct path coefficient (*P_iy_*) [35]. The calculation of the path coefficient (*P_iy_*) is the hardest part in general terms [36].

The path analysis in this study was conducted by using the data obtained at the intermediate microwave intensity of 8 W/g. As the drying time was 11 min, and there were four independent variables and one dependent variable in this study, the sample size was 55, which is a small sample test. Thus, the Shapiro-Wilk test was selected for the normality test. The temperature, moisture content, drying time, and radius of the berry puree were independent variables, and the anthocyanin content of the berry puree was the dependent variable. After the above data was input into SPSS software, the commands of Analyze, Regression, and Linear were chosen successively. The Method was specified as Stepwise, and then Descriptive was selected in Statistics. The standardized coefficients of the linear regression equation and Pearson calculation results were obtained in the calculation results. As the independent variables were gradually brought into the regression equation, the determination coefficient *R*^2^ increased gradually. The growth of *R*^2^indicated that the effect of the introduced independent variables on the anthocyanins content increased.

As presented in Table 1, the correlation coefficient increased significantly with the introduction of moisture content, while there was no obvious growth with the introduction of drying time and radius. The final determination coefficient of regression *R*^2^ was 0.976 after the introduction of four independent variables. The remaining factor e = $\sqrt{1-R^{2}}$ was 0.155, which indicated that there exists some other factors that cause the degradation of anthocyanin during the MFD process in addition to these four independent variables. The non-thermal effect that occurred during the microwave drying might be one of the reasons [37]. In addition, other ingredients in the berry puree also have an impact on the stability of anthocyanins, such as the sugars, organic acids, and so on [38]. As shown in Table 2, all of the influencing factors were significant to anthocyanin content, except for the radius (*p* < 0.001). Path analysis was performed after removing the non-significant factor.

From Table 3, the temperature and drying time both had negative correlations with the anthocyanin content, while the moisture content was positively correlated with the anthocyanin content. The direct path coefficient of temperature on anthocyanin content was −0.167, while the indirect path coefficient of through the moisture content on anthocyanin content was −0.921. The direct path coefficient of moisture content on anthocyanin content was 0.986, and the sum of the indirect path coefficient was −0.001, which indicated that a higher moisture content can protect the anthocyanins in berry puree. As the anthocyanins are water-soluble pigments, an aquatic environment is more suitable for them [39]. The drying time had a negative correlation with anthocyanin content, and it was mainly indirect effects through the moisture content.

#### 3.2.2. The effect of the Ratio of Moisture Content to Temperature (M/T) on Anthocyanin Degradation

**Figure 7:**
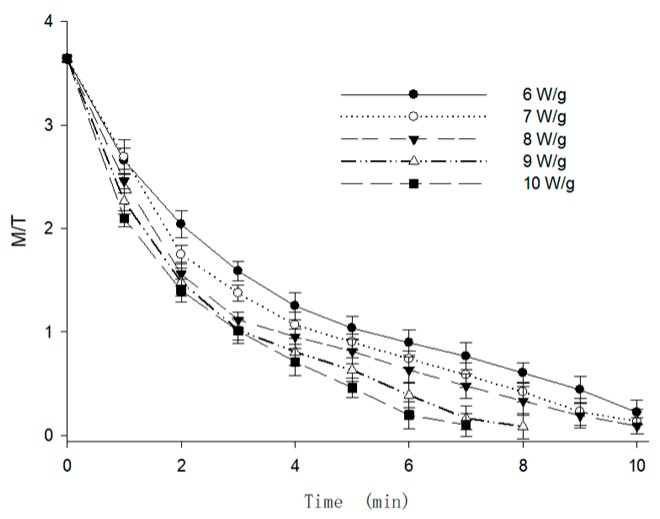
Changes in the moisture content to temperature ratio (*M/T*) of berry puree under different microwave intensities during microwave assisted foam-mat drying (MFD).

Based on the path analysis results, the temperature and moisture content were found to be the two most important factors affecting the stability of anthocyanins during MFD. As the drying process was always combined with the changes of moisture content and temperature, the ratio of moisture content to temperature (*M/T*) was proposed first as a comprehensive evaluation index to analyze the relationship between the anthocyanin degradation and the drying process parameters. As demonstrated in the drying characteristics illustrated in Section 3.1, the ranges of moisture content and temperature during the MFD were 15–90%, and 25–125 °C, respectively. Therefore, the value of *M/T* was decreased from the initial value of 3.6. Figure 7 shows the change of *M/T* with the drying time under different microwave intensities. As shown in the fitting curve in Figure 7, the *M/T* was expressed as the function of the microwave intensity and drying time in Equation (3).

(3) $$\frac{M}{T}=0.1375+3.445e^{-0.04792P\cdot t}$$

where *P* is the microwave intensity (W/g) and *t* is the drying time (min).

**Figure 8:**
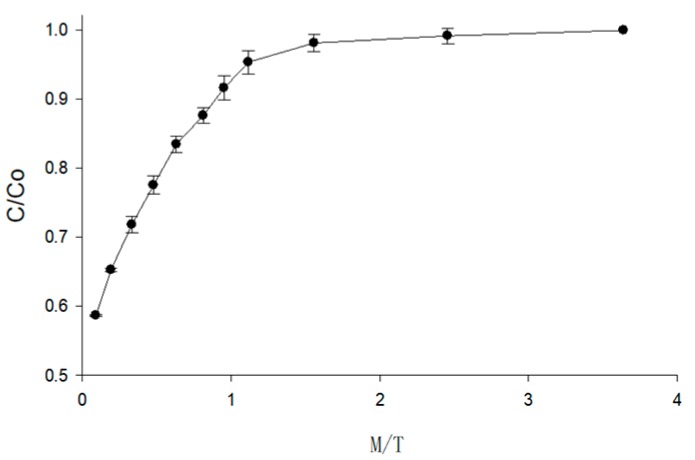
The changes of the anthocyanin retention ratio of berry puree with the *M/T*.

The effect of *M/T* on the anthocyanin retention ratio is shown in Figure 8. The curve of the anthocyanin retention ratio was gentle for *M/T* values in the range between 0.96 and 3.6, which indicates that the anthocyanin in berry puree seldom degraded during this period. When the *M/T* was below 0.96, the slope of the curve increased sharply. Corresponding to the trend of moisture content and temperature changes in MFD, in the middle of the foaming drying stage the temperature was around 75 °C and the moisture content decreased rapidly. These environmental conditions were detrimental to the existence of anthocyanins. The anthocyanin retention ratio decreased exponentially when the *M/T* was below 0.125 in the rapid heating stage. The temperature of the berry puree was higher than 100 °C and the moisture content was lower than 25% at that time. The *M/T* was a representative index, which could represent the drying stage intuitively. In order to obtain the maximum retention of anthocyanin in dried berry products, the drying conditions should be controlled to ensure that the *M/T* value remains larger than 0.96. By fitting the data in Figure 8, the relationship between the *M/T* and the anthocyanin retention ratio is shown in Equation (4).

(4) $$\frac{C}{C_{0}}=1.009-0.4993e^{-1.694\frac{M}{T}}\;(R^{2}=\;0.9958)$$

where *C* is the anthocyanin content at a certain point in time (mg/g) and *C*_0_ is the initial anthocyanin content (mg/g).

### 3.3. Degradation Kinetics Analysis of Anthocyanins during MFD

The amount of anthocyanin degradation can be related to the drying conditions by using *M/T* as an intermediate transfer function. In order to further clarify the causes of anthocyanin degradation, the degradation kinetics of *M/T* on the anthocyanins was analyzed. According to the determination coefficients, the degradation of the anthocyanins in berry puree during MFD followed the first-order reaction kinetics of Equation (5). Equation (6) was obtained by integrating Equation (5).

(5) $$C=C_{0}e^{\left(-kt\right)}$$

(6) $$\ln\frac{C}{C_{0}}=-kt$$

where *k* is the reaction rate (min^−1^) and *t* is the drying time (min).

As Equation (6) illustrates, the *ln(C/C*_0_*)* and drying time *t* accorded with a linear relationship. When plotting the change of anthocyanins contents and drying time, the reaction rate constant *k* was obtained by reading the slope of the curve. The reaction rate constant *k* is an expression of the reaction rate; it is a function of activation energy and temperature [40]. Their relationship can be expressed by the *Arrhenius* equation as shown in Equation (7).

(7) $$k_{T}=A_{T}\cdot \exp\left(-\frac{E_{a}}{R_{g}T}\right)$$

where *k_T_* is the reaction rate constant at temperature *T* (min^−1^), *A_T_* is the *Arrhenius* constant (min^−1^), *E_a_* is the activation energy (kJ/mol) and *R_g_* is the ideal gas constant, 8.314J/(mol·K).

Substituting the reaction rate constants under different temperature conditions into the logarithm of Equation (7), the activation energy of the anthocyanin degradation were obtained. A higher activation energy indicates that the reaction required more energy. In this study, a higher activation energy indicates a more difficult degradation of anthocyanin, which is beneficial for the retention of anthocyanin during microwave drying.

The *Eyring* equation was established based on the *Arrhenius* equation to describe the effects of pressure on bioactive ingredients [41]. The function between the reaction rate constant *k_M_*, reaction heat *H_a_*, and the moisture content *M* under different moisture contents could be obtained by modifying the *Eyring* equation as shown in Equation (8).

(8) $$k_{M}=A_{M}\cdot \exp\left(\frac{-H_{a}\cdot M_{H_{2}O}\cdot M}{R_{g}\cdot T}\right)$$

where *k_M_* is the reaction rate constant at moisture content *M* (min^−1^), *A_M_* is the *Eyring* constant (min^−1^), *H_a_* is the reaction heat (kJ/kg), and *M_H_2_O_* is the molar mass of water, 18 g/mol.

#### 3.3.1. Preheating Drying Stage

Based on the characteristics of different stages of MFD, the anthocyanin degradation kinetic analysis model was established in different stages.

The preheating stage was considered to be an energy storage process, where the temperature of the berry puree increased gradually, while the moisture content decreased slightly. Since the change of berry puree in this drying stage was mainly caused by the variation of temperature, the activation energy can be calculated using the *Arrhenius* Equation (9).

(9) $$k_{T}=A\cdot \exp[\frac{-E_{a}}{R_{g}}\cdot (\frac{1}{T}-\frac{1}{T_{0}})]$$

**Figure 9:**
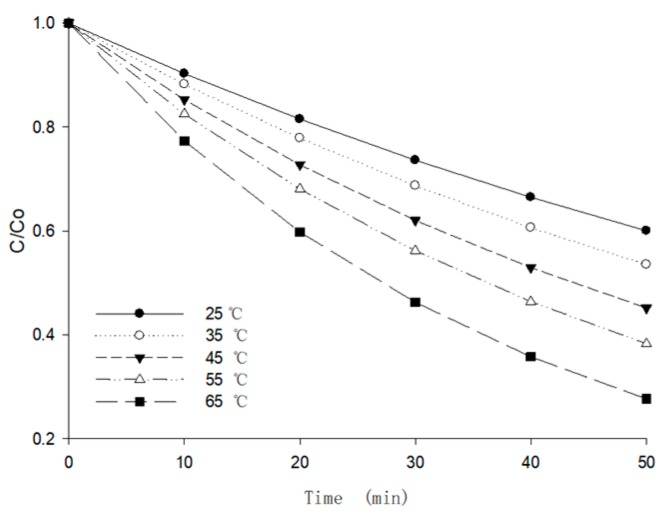
The effects of temperature on the anthocyanin retention ratio of berry puree.

In order to be close to the conditions of the real drying stages, the moisture content was kept at 90%, then measured the anthocyanin retention ratio at different temperatures (25–65 °C by interval of 10 °C). As shown in Figure 9, the anthocyanin retention ratio had a negative correlation with temperature in high moisture conditions. The maximum anthocyanin degradation ratio was 72.34% at 65 °C at 50 min (*p* < 0.05). The reaction rate constant of anthocyanin was obtained by fitting the data in Figure 9. By fitting to Equation (9) the activation energy in this stage could be calculated (*p* < 0.05), and the results are illustrated in Table 4.

#### 3.3.2. Foaming Drying Stage

Most of the dehydration in the berry puree happened at the foaming drying stage in the MFD process. As the temperature fluctuated in the range of 75–85 °C, the change in berry puree was mainly caused by the variation in the moisture content at this stage. Therefore, the reaction heat of the anthocyanins can be calculated by the modified *Eyring* Equation (10).

(10) $$k_{M}=A_{M}\cdot \exp[\frac{-H_{a}\cdot M_{H_{2}O}}{R_{g}\cdot T}\cdot (M-M_{0})]$$

**Figure 10:**
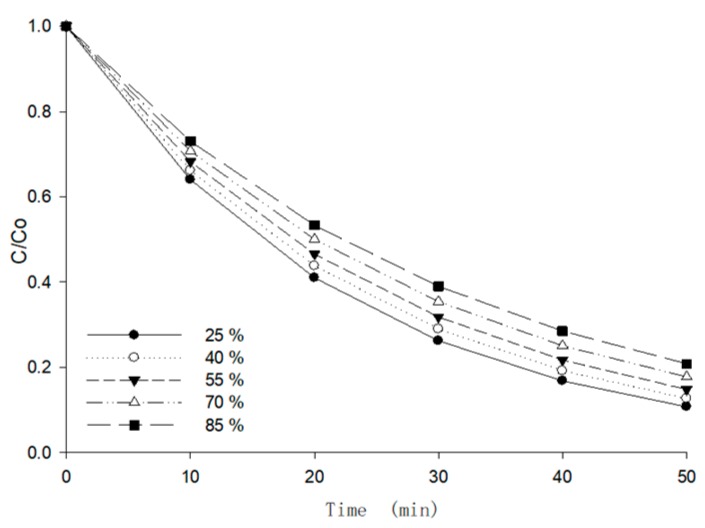
The effects of the moisture content on the anthocyanin retention ratio of berry puree.

The experiments were designed to study the effect of moisture content on the degradation of anthocyanin at a temperature of 75 °C, and then to measure the anthocyanin retention ratio at moisture contents from 25% to 85% (interval of 15%). As shown in Figure 10, the anthocyanin retention ratio had a positive correlation with the moisture content at 75 °C (*p* < 0.05). The reaction rate constant and reaction heat in this stage are shown in Table 5. The minus before the value of *H_a_* in Table 5 indicates that the degradation rate decreased with the increase in moisture content.

#### 3.3.3. Rapid Heating Stage

In the rapid heating stage, the moisture content decreased from 25% to 15%, while the temperature increased dramatically. In order to be familiar with the circumstances of the real drying stage, the moisture content of the berry puree was set to 25%, and then the anthocyanin retention ratio was measured at different temperatures (80–120 °C by interval of 10 °C). In this drying stage, the degradation process can be described by the combined *Arrhenius-Eyring* Equation (11).

(11) $$k=k_{ref}\cdot \exp[\frac{-E_{a}}{R}\cdot (\frac{1}{T}-\frac{1}{T_{0}})]\cdot \exp[\frac{-H_{a}\cdot M_{H_{2}O}}{R\cdot T}\cdot (M-M_{0})]$$

where *k_ref_* is the *Arrhenius-Eyring* constant (min^−1^).

**Figure 11:**
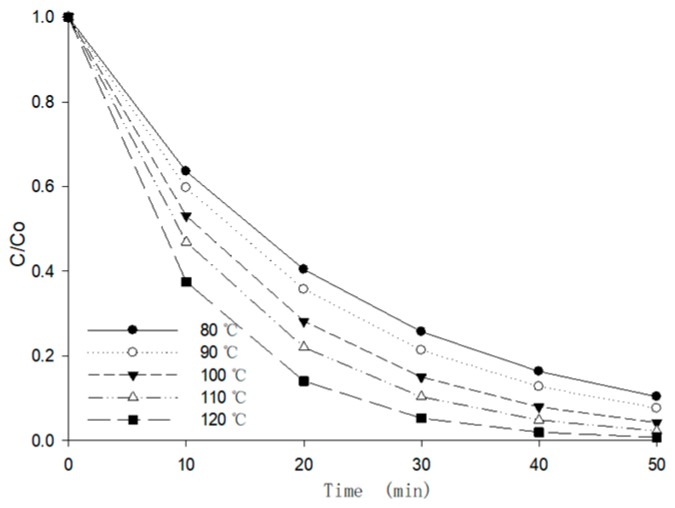
The effects of temperature on the anthocyanin retention ratio of berry puree.

As shown in Figure 11, the anthocyanin retention ratio in berry puree dropped sharply, and all the degradation ratios were over 50% within 16 min (*p* < 0.05). The anthocyanin degradation was more than 80% if the temperature exceeded 100 °C for 20min due to the interaction of low moisture content and harsh temperature. Although the drying time of the MFD process was short, the duration of this condition should be avoided. The process might be improved by intermittent drying, adding tempering steps, or introducing vacuum conditions. The degradation rate constants, activation energy, and reaction heat of the anthocyanin are shown in Table 6.

### 3.4. A Simulation Model of Anthocyanin Degradation during MFD

The anthocyanin degradation occurred simultaneously with the increase of temperature and the decrease of moisture content during the MFD process. A multiphysics simulation model considering the electromagnetic field, temperature field, and concentration field was built. This will provide an intuitive reference for the research of anthocyanin degradation.

#### 3.4.1. Model Assumptions

The following assumptions were included in this model. The berry puree was assumed to be a uniform and stable continuous medium. The microwave was incident from the edges of the container. The effects of other influencing factors on anthocyanins, such as light, oxygen, non-thermal effects, and so on, were ignored. The container carrying the berry puree rotated with the turntable as a whole.

#### 3.4.2. Geometric Model

**Figure 12:**
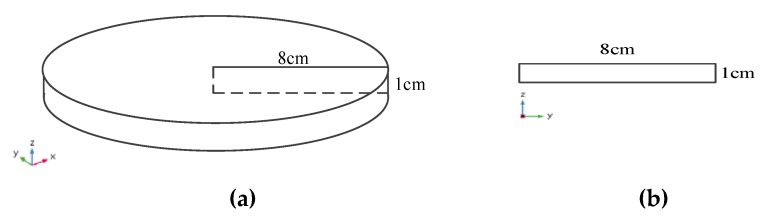
Geometry of the berry puree: (**a**) 3D structure; (**b**) Simplified two-dimensional structure.

The berry puree loaded in the glass tray can be considered to be a three-dimensional cylinder structure (Figure 12a). As the drying status in each cross section of the cylinder was the same, thus the simulation of anthocyanin degradation was conducted with a simplified two-dimensional rectangle structure (Figure 12b). Then the calculation time of the simplified model was reduced, while the accuracy of the model was unchanged.

#### 3.4.3. Governing Equation

The excitation of the microwave workstation was through the bottom of a rectangular waveguide. The volumetric heat of the berry puree generated by absorbing the microwave energy in a unit volume was calculated using Equation (12).

(12) $$Q(x,y,z,t)=2\pi f\varepsilon_{0}\varepsilon^{\prime \prime }E^{2}$$

where *Q* is the volumetric heat (J/m^3^∙s), *f* is the microwave frequency 2.45 (GHz), *ε*_0_ is the vacuum permittivity, 8. 85 × 10^−12^ (F/m), *ε″* is the dielectric loss factor, and *E* is the electric field intensity (V/m).

The dynamic changes of the thermal characteristics parameters and the dielectric characteristics of the berry puree were considered in this model. The energy conservation of the MFD process was established in Equation (13), including the heat conduction, heat convection, evaporative cooling, and the endotherm of degradation.

(13) $$Q(x,y,z,t)=\rho C_{p}\frac{\partial T}{\partial t}+K\Delta^{2}T+\lambda \frac{\partial M_{E}}{\partial t}+\left(C_{0}-C\right)\cdot E_{a}$$

where *ρ*—is density of the berry puree (kg/m^3^), *C_P_* is the specific heat capacity of the berry puree (J/(kg·K)), *K*—is the thermal conductivity of berry puree (W/(m·K)), *λ* is the latent heat of vaporization (2.26 × 10^3^ kJ/kg), and *M_E_* is the amount of evaporation (kg).

The content of anthocyanins in berry puree can be calculated according to Equation (4). The temperature *T* and the moisture content *M* of berry puree are variables in Equation (4), which can be solved by the energy conservation Equation (13).

#### 3.4.4. Initial Conditions and Meshing

**Figure 13:**
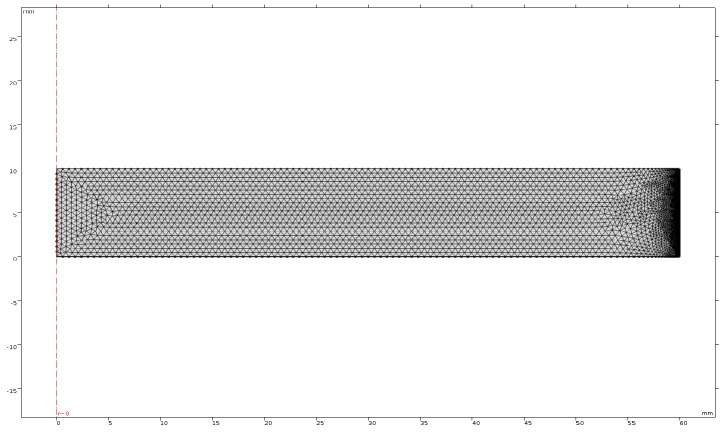
The meshing of the anthocyanin degradation model.

The initial conditions for the variables are listed in Table 7 [42]. The initial anthocyanin content of the berry puree was 57.83 mg/g. The volumetric heat generated by the microwave was used as the heat source and substituted into the two-dimensional model as the energy source. The entire domain was meshed into triangle elements. Boundary layers were created at the edges of berry puree to improve the convergence (Figure 13).

#### 3.4.5. Experimental Validation

The simulated anthocyanin contents were compared with the experimental results using the root mean square errors (*RMSE*) given by Equation (14).

(14) $$RMSE=\sqrt{\frac{1}{n}\sum_{i=1}^{n}{\left(X_{s}-X_{e}\right)}^{2}}$$

where *X_s_* and *X_e_* are, respectively, the simulated and experimental anthocyanin content (mg/g) and *n* is the total number of time steps recorded during the heating.

**Figure 14:**
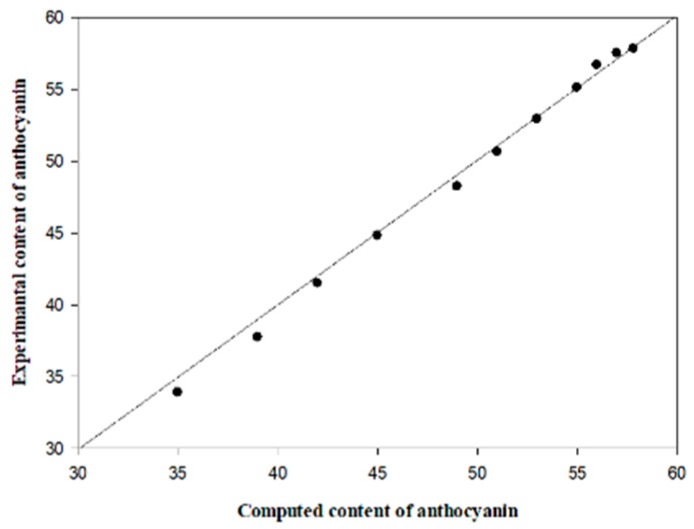
A comparison between the simulated and experimental anthocyanin content.

As shown in Figure 14, the numerical simulations were in good agreement with the experimental results in general. The *RMSE* between the simulated and experimental results was less than 5%, which indicated that the model can accurately reflect the degradation of anthocyanins during the MFD process.

#### 3.4.6. Simulated Anthocyanin Degradation Rates in Different Drying Stages

The degradation rate (mol/m^3^·s) is an important indicator which characterizes the amount of nutrient degradation in a unit volume per unit time. An increase in the degradation rate indicates that the nutrient is more prone to degradation, which is a desired sign in the biochemical reaction of some pollutants [43]. However, in this study, low degradation rate values were desired.

**Figure 15:**
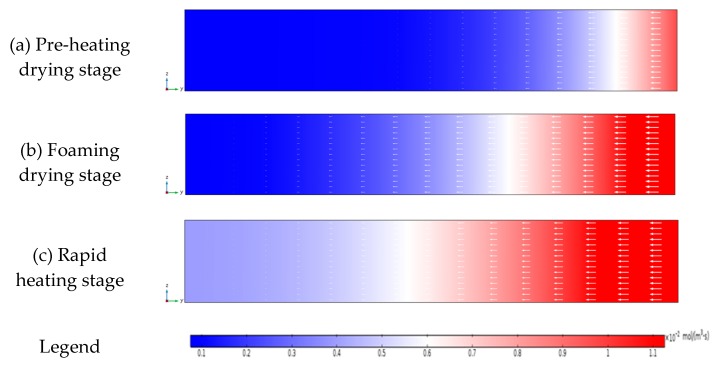
The distribution of the anthocyanin degradation rates during different drying stages.

As shown in Figure 15, there was a clear gradient of the anthocyanin degradation rates during three drying stages. The degradation rate decreased along the microwave transmission direction, and the higher degradation area (red area) moved gradually from the edge toward the center. The results showed that the anthocyanin degradation rate was consistent with the change of temperature [25].

#### 3.4.7. The Simulation of Anthocyanin Content at Different Microwave Intensities

**Figure 16:**
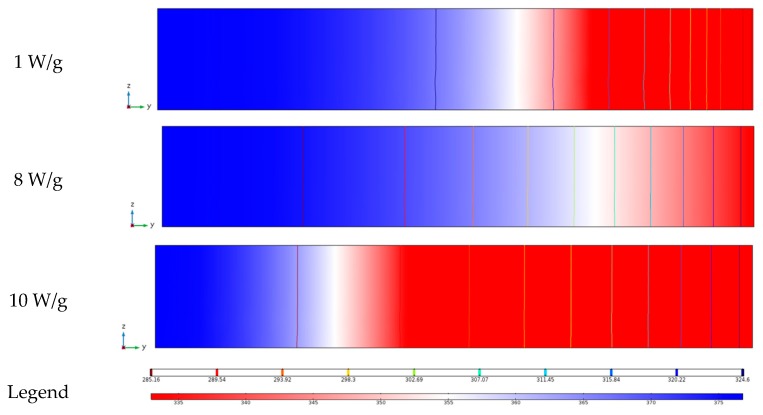
The distribution of the anthocyanin content and contours in the berry puree under different microwave intensities.

The simulated anthocyanin content in dried berry products at the microwave intensity of 1 W/g, 8 W/g and 10 W/g are presented in Figure 16. The residual anthocyanin content was at the minimum at a microwave intensity of 10 W/g. This result was attributed to the degradation of anthocyanin caused by high temperature under a high microwave intensity. As the drying time was prolonged as the result of low drying efficiency, the anthocyanin degradation was also severe at the microwave intensity of 1 W/g.The anthocyanin retention in the dried products was at the maximum at a microwave intensity of 8 W/g, because the heat efficiency and drying time were both at the optimal levels for drying berries. The content of anthocyanin loss was higher at the edges than at the center. The difference of anthocyanin content between the edges and the center was 1.56 × 10^2^ mol/m^3^ at 1 W/g and 0.85 × 10^2^ mol/m^3^ at 10 W/g. The increase in microwave intensity was helpful in reducing the difference of anthocyanin degradation among different sampling points, because the drying efficiency was improved.

## 4. Conclusions

In this study, the effects of different variables on the degradation of anthocyanin in berry puree during the MFD process were investigated. The anthocyanins in berry puree were relatively stable during the pre-heating drying stage and the foaming drying stage; they were mainly degraded in the rapid heating stages. Path analysis was conducted between the temperature, moisture content, drying time, radius, and the anthocyanin retention ratio. The direct path coefficient of the moisture content on anthocyanins was 0.985, and the direct path coefficient of the temperature on anthocyanins was −0.933. The *M/T* was first proposed as a comprehensive evaluation factor to estimate the degradation of anthocyanins during MFD. In order to maintain the stability of anthocyanins, the drying conditions should be controlled to ensure that the *M/T* is larger than 0.96. The analysis of the degradation kinetics was implemented in stages according to the drying characteristics. The activation energy of the anthocyanin degradation was 4.756 kJ/mol in the preheating drying stage, and it was 2.569 kJ/mol in the rapid heating stage. The simulation results were in agreement with the experimental results in general. The simulated anthocyanin degradation rate was consistent with the changes of *M/T*. The MFD process for drying berries should be conducted at an optimal microwave intensity to minimize the degradation of anthocyanins.

## Figures and Tables

**Table 1:** The results of the regression analysis of anthocyanin content.

Model	*R*	*R* ^2^	Adjusted *R*^2^	Standard Error Estimation
1	0.933 ^**a^	0.871	0.869	5.419
2	0.986 ^**b^	0.972	0.971	2.525
3	0.987 ^**c^	0.973	0.972	2.516
4	0.988 ^*d^	0.976	0.974	2.391

** Significance at the 0.001 level. * Significance at the 0.01 level. a Regression analysis of temperature, and anthocyanins content. b Regression analysis of temperature, moisture content, and anthocyanins content. c Regression analysis of temperature, moisture content, drying time, and anthocyanins content. d Regression analysis of temperature, moisture content, drying time, radius, and anthocyanins content.

**Table 2:** The correlation coefficients of the influencing factors on anthocyanin content.

Parameters	Anthocyanin *Y*_1_	Temperature *X*_1_	Moisture Content *X*_2_	Drying Time *X*_3_	Radius *X*_4_
***Y*_1_**	1.000	−0.933 ^**^	0.986 ^**^	−0.961 ^**^	−0.091
***X*_1_**		1.000	−0.934^**^	0.964 ^**^	0.102
***X*_2_**			1.000	−0.975 ^**^	−0.117
***X*_3_**				1.000	0.000
***X*_4_**					1.000

** Significance at the 0.001 level. “−” The minus implies two factors have a reverse increasing tendency.

**Table 3:** Path analysis results of the influencing factors on anthocyanin content.

Parameters	*R*	Direct Path Coefficient	Indirect Path Coefficient			
			Sum	*X*_1_	*X*_2_	*X*_3_
***X*_1_**	−0.933 ^**^	−0.167 ^**^	−0.766	—	−0.921	0.155
***X*_2_**	0.985 ^**^	0.986 ^**^	−0.001	0.156	—	−0.157
***X*_3_**	−0.961 ^**^	0.161 ^**^	−0.922	−0.161	−0.961	—

** Significance at the 0.001 level.

**Table 4:** The degradation rate constants and activation energy of anthocyanin in berry puree at different temperatures.

*T* (°C)	25	35	45	55	65
***k*** (min^−1^)	0.0102 ± 0.0021	0.0125 ± 0.0012	0.0159 ± 0.0037	0.0192 ± 0.0033	0.0257 ± 0.0113
*R* ^2^	0.9685	0.9761	0.9623	0.9864	0.9763
Standard Error Estimation	0.0183	0.0208	0.0062	0.0160	0.0023
***E_a_*** (kJ/mol) (95% EI)	4.756 (3.908, 5.604)				
*R* ^2^	0.9825				

**Table 5:** Degradation rate constants and reaction heat of anthocyanin in berry puree in different moisture content.

*M* (%, *w.b.*)	25	40	55	70	85
***k*** (10^−2^min^−1^)	0.0446 ± 0.0682	0.0413 ± 0.0048	0.0382 ± 0.0030	0.0346 ± 0.0039	0.0314 ± 0.0009
*R* ^2^	0.9767	0.9846	0.9637	0.9721	0.9687
Standard Error Estimation	0.0065	0.0127	0.0096	0.0419	0.0274
***H_a_*** (kJ/kg) (95% EI)	−0.658 (0.5943, 0.7217)				
*R* ^2^	0.9736				

**Table 6:** The degradation rate constants, activation energy and reaction heat of anthocyanins in berry puree at different temperatures.

*T* (°C)	80	90	100	110	120
***k*** (10^−2^min^−1^)	0.0453 ± 0.0359	0.0515 ± 0.0296	0.0633 ± 0.0044	0.0757 ± 0.0226	0.0982 ± 0.2248
*R* ^2^	0.9982	0.9683	0.9876	0.9782	0.9754
Standard Error Estimation	0.1057	0.0862	0.0953	0.1349	0.7361
***E_a_*** (kJ/mol) (95% EI)	2.569 (1.721, 3.417)				
*R* ^2^	0.9663				
***H_a_*** (kJ/kg) (95% EI)	−1.257 (−1.193, −1.321)				
*R* ^2^	0.9741				

**Table 7:** The input parameters of berry puree used in the simulation model.

Parameter	Value	Unit
**Initial temperature** (*T*_0_)	25	°C
**Initial moisture content** (*M*_0_)	90	%
**Dielectric constant** (*ε*)	47.29 + 5.1*M* − 1.45*T* + 3.30*D* + 0.57*MT* + 0.56*M*^2^ − 2.64*T*^2^	
**Dielectric loss** (*ε″*)	9.13 + 1.11*M* − 0.49*T* + 0.98*D*	
**Density** (**ρ**)	600	kg/m^3^
**Specific heat capacity** (*C_P_*)	2.06 + 0.16*M* − 0.078*T* + 0.044 *MT*−0.066*TD* − 0.12*T*^2^	kJ/kg·K
**Thermal conductivity** (*k*)	0.31 + 0.026*M* − 8.777 × 10^−3^*T* + 0.04*D*	W/m·K
